# The Development and Validation of the Online Shopping Addiction Scale

**DOI:** 10.3389/fpsyg.2017.00735

**Published:** 2017-05-16

**Authors:** Haiyan Zhao, Wei Tian, Tao Xin

**Affiliations:** ^1^Faculty of Psychology, Beijing Normal UniversityBeijing, China; ^2^Beijing Education Examinations AuthorityBeijing, China; ^3^Collaborative Innovation Center of Assessment toward Basic Education Quality at Beijing Normal UniversityBeijing, China

**Keywords:** online shopping addiction, behavioral addiction, internet addiction, compulsive buying, scale development

## Abstract

We report the development and validation of a scale to measure online shopping addiction. Inspired by previous theories and research on behavioral addiction, the Griffiths's widely accepted six-factor component model was referred to and an 18-item scale was constructed, with each component measured by three items. The results of exploratory factor analysis, based on Sample 1 (999 college students) and confirmatory factor analysis, based on Sample 2 (854 college students) showed the Griffiths's substantive six-factor structure underlay the online shopping addiction scale. Cronbach's alpha suggested that the resulting scale was highly reliable. Concurrent validity, based on Sample 3 (328 college students), was also satisfactory as indicated by correlations between the scale and measures of similar constructs. Finally, self-perceived online shopping addiction can be predicted to a relatively high degree. The present 18-item scale is a solid theory-based instrument to empirically measure online shopping addiction and can be used for understanding the phenomena among young adults.

## Introduction

Initial definitions of addiction focused on drug ingestion or intake of substances (Walker, 1989; Rachlin, 1990). Some behaviors of this kind can be regarded as substance addiction. Other behaviors that do not involve drug ingestion also have the potential for addiction, albeit with psychological and physiological correlates similar with drug ingestion (Shaffer et al., 2004). Research on the non-substance-related or behavioral addiction is growing. Examples of such addiction include game playing (e.g., Fisher, 1994; Lemmens et al., 2009), gambling (Griffiths, 1995; Brand et al., 2005), overeating (Orford, 2001), exercise (Adams and Kirkby, 2002; Berczik et al., 2012), internet use (e.g., Young, 1998; Beard, 2005), shopping (Clark and Calleja, 2008; Davenport et al., 2012), cellphone use (Rutland et al., 2007; Chóliz, 2010), and work (Andreassen et al., 2010; Andreassen, 2014).

With the popularity of the wired lifestyle (Bellman et al., 1999), online shopping addiction (OSA) has begun to appear as a new behavioral addiction. According to Rose and Dhandayudham (2014), OSA may have negative influences not only on an individual's daily life and social life, but also on their economic status. Consequently, the diagnosis, intervention, and treatment of OSA are of great importance. Thus, a reliable and valid instrument to measure OSA is essential. To operationalize OSA, it is helpful to consider some similar constructs, such as internet addiction (IA) and compulsive buying (CB).

Over the last 15 years, there is debate about whether IA is a genuine addiction (Griffiths and Pontes, 2014). Since “Gambling Disorder” has been re-classified as a disorder of addiction instead of impulse control in the latest edition of the Diagnostic and Statistical Manual of Mental Disorders (DSM-5) (American Psychiatric Association, 2013), that re-classification suggests considering IA as a genuine addiction. According to Davis (2001), IA can be classified into specific and generalized types depending on the target of the behavior. The former IA uses the internet for particular purposes, such as online gaming, gambling, social networking, etc., while the latter IA had no specific aims.

As far as OSA is concerned, many researchers hold that OSA can be classified into the category of specific IA (Brand et al., 2014; Griffiths and Szabo, 2014; Laconi et al., 2015; Montag et al., 2015; Pontes et al., 2015). Griffiths (2000) argued that it is important to distinguish between addictions on the internet and addictions to the internet. Specifically, many people spending excessive time on the internet are not addicted to the medium itself, but use the medium to actualize other addictions (Pontes et al., 2015). From this perspective, OSA should be a specific type of IA.

CB refers to a tendency toward long-term, repeated buying behavior, which has become the individual's primary response to negative events and emotions (O'Guinn and Faber, 1989; Black, 2007; Müller et al., 2015; Trotzke et al., 2015). Many researchers regard CB as a behavioral addiction (Demetrovics and Griffiths, 2012; Lo and Harvey, 2012; Starcke et al., 2013; Rose and Dhandayudham, 2014), while others emphasize that a typical behavioral addiction involves much time spent in thinking about engaging in the behavior and is therefore characterized by intense preoccupation (Sussman et al., 2010). Although, OSA and CB are rather similar in both external manifestation and internal features, there may still be subtle differences between them, such that OSA may be confined to the internet, while CB has no such restriction.

### Assessment of online shopping addiction

To the best of our knowledge, no specialized instruments for OSA exist, although some relevant instruments need to be mentioned. The first is the Bergen Shopping Addiction Scale (Andreassen et al., 2016), which was designed to measure the core criterion and components of shopping addiction. The scale consists of 28 items, four for each of the seven addiction criteria listed in the Diagnostic and Statistical Manual of Mental Disorders (DSM-IV-TR; American Psychiatric Association, 2000). The content of the items reflect contemporary shopping habits and the scale show good validity and reliability. Other relevant instruments include those used by researchers examining OSA as a specific IA. For instance, to assess OSA, the corresponding subscale of the Shorter PROMIS questionnaire (Christo et al., 2003) was modified by adding the terms “internet” or “online.” The resulting ten-item scale proved to be reasonably reliable (Laconi et al., 2015). In another instance, Montag et al. (2015) used the short version of the Gaming Addiction Scale (Lemmens et al., 2009) as a blueprint and constructed several specific IA scales including OSA by exchanging the word “game” in each item with each specific form of IA. The resulting seven-item scale also had high consistency across different samples.

All the instruments mentioned above followed the common practice in behavioral addiction, in which instruments were constructed based on certain factor models, such as the six-factor model (Brown, 1993; Griffiths, 1996) or the seven-addiction criteria of DSM-IV (American Psychiatric Association, 2000). Although, the authors of these instruments claim or imply the existence of a specific IA, they employ a similar construct structure to the generalized IA. The particularity of instruments is item wordings, whether a specific or generalized IA they are designed for. The particularity of specific IA was never examined or resolved in the content or expressions of items, let alone the relationship and distinction between specific and generalized IA. Nevertheless, there are instruments constructed based on different structures, such as the Facebook Addiction Scale (Torsheim et al., 2012) and the Game Addiction Scale (Lemmens et al., 2009). The former was based on a one-factor solution and the latter was on a second-order structure. Both these scales exhibited sound reliability and validity, which implied that other structures might also be plausible for these specific IA.

The aim of the present study was to construct a specialized instrument for OSA. We sampled college students as participants, because in China, individuals of this age are independent of their parents and they are skillful in using the internet. Previous research also suggests that the similar disorder, CB, usually has an onset in one's 20s and turns into a chronic disorder in their later years (Black, 2007).

### Development of the online shopping addiction scale

In the present study, we view OSA as a specific type of IA. Referring to the definitions of other behavioral addiction (Griffiths, 2005), we defined OSA as a tendency of excessive, compulsive and problematic shopping behavior via the internet that results in consequences associated with economic, social, and emotional problems. Addictive shoppers still fail to control their excessive online shopping behaviors despite problematic consequences.

During the development of the OSA scale, we employed the six-factor model of behavioral addiction (Brown, 1993; Griffiths, 1996, 2002). This model holds that the following six elements are necessary for operational definitions of addictive behaviors: salience, mood modification, tolerance, withdrawal, conflict, and relapse. Salience means that addiction behaviors have become the most important activity in addicts' lives, preoccupying their thoughts, dominating their cravings, and demonstrating an excessive occurrence. Mood modification refers to the subjective experience of conducting addictive behaviors, e.g., feeling high, buzz or exciting, quiet, released, numb or even depressed after fulfillment. Tolerance means that in order to achieve the effects equal to that in the past, addicts have to increase the amount of the activities. Withdrawal indicates the unpleasant sensation and/or physiological reaction after the addictive behaviors are cut off or restricted. Conflict indicates the interpersonal conflict between individuals and others together with the intrapsychic conflict within individuals. Relapse refers to the tendency of returning to the original behavioral modes after dropping or restricting addictive behaviors; the addiction will burst into the most severe degree after relapse (Brown, 1993; Griffiths, 1996, 2005).

In practice, we began by developing a pool of possible items, reflecting these six factors. When constructing the preliminary items, we also referred to several behavioral addiction or compulsive buying instruments (e.g., Christo et al., 2003; Lemmens et al., 2009; Torsheim et al., 2012; Müller et al., 2015; Andreassen et al., 2016). We considered the circumstances of online shopping and adapted the impacts of other behavioral addiction to the context of online shopping. Three linguistic specialists and three researchers specializing in psychometrics reviewed the raw items. Then we modified the items elaborately based on their comments and data from pre-tests. In all, the review and modification process took three rounds. The preliminary scale contained 27 items, four or five for each of the six components. Each item included a sentence about the strength, effect, and internal or external influence of online shopping. The final scale was listed in Table 1 together with the component that each item belonged to.

**Table 1:** **Mean scores, standard deviation, measures of distribution, and the corrected item-total correlation for the18-item online shopping addiction scale based on the exploratory sample**.

**Subscales**	**Item**	**Item content**	**M**	***SD***	**Skewness**	**Kurtosis**	**CITC**
Salience	S1	When I am not shopping online, I keep thinking about it	3.44	1.10	−0.61	−0.42	0.45
	S2	I frequently think about how to spare more time or money to spend in online shopping	3.27	1.17	−0.23	−0.84	0.53
	S3	Online shopping is important for my life	3.80	1.03	−0.89	0.30	0.38
Tolerance	T1	Recently, I have an urge to do more and more online shopping	2.38	1.18	0.47	−0.86	0.55
	T2	I spend more and more time in online shopping	2.13	1.13	0.75	−0.43	0.56
	T3	Recently I often shop online unplanned	2.62	1.29	0.10	−1.32	0.53
Mood modification	M1	When I feel bad, online shopping can make me feel good	3.28	1.11	−0.33	−0.61	0.54
	M2	When I am feeling down, anxious, helpless or uneasy, I shop online in order to make myself feel better	2.23	1.25	0.60	−0.94	0.45
	M3	Online shopping can help me to temporarily forget the troubles in real life	2.32	1.22	0.48	−0.98	0.57
Withdrawal	W1	When I can't do online shopping for certain excuses, I will get depressed or lost	2.19	1.14	0.67	−0.58	0.63
	W2	Life without online shopping for some time would be boring and joyless for me	2.23	1.22	0.66	−0.74	0.68
	W3	I will feel restless or depressed when attempting to shop online but unable to achieve	2.46	1.22	0.34	−1.11	0.55
Relapse	R1	I have tried to cut back or stop my online shopping, but failed	2.16	1.09	0.77	−0.22	0.59
	R2	I have decided to do online shopping less frequently, but not managed to do so	2.06	1.03	0.77	−0.26	0.59
	R3	If I cut down the amount of online shopping in one period, and then start again, I always end up shopping as often as I did before	1.86	1.04	1.09	0.32	0.67
Conflict	C1	My productivity for work or study has decreased as a direct result of online shopping	1.68	0.88	1.35	1.57	0.45
	C2	I have once quarreled with my parents for my online shopping	1.42	0.83	2.31	5.26	0.25
	C3	I have cut off my time with parents and friends for my online shopping	1.53	0.79	1.69	2.81	0.52

*M, Mean; SD, Standard Deviation; CITC, Corrected Item-Total Correlation*.

## Methods

### Participants and sampling

We collected three groups of participants from about 30 colleges in China for the purpose of scale development and validation. Sample 1, the exploratory sample, consisted of 999 students (744 female); their age varied between 18 and 28 (Mean = 21.05; *SD* = 1.87). Sample 2, the confirmatory sample, consisted of 854 students (575 female); their age ranged from 18 to 28 (Mean = 21.36; *SD* = 2.04). Sample 3, the validation sample, consisted of 328 students (159 female); their age ranged from 18 to 28 (Mean = 21.79; *SD* = 2.25).

### Measures

#### Demographic questionnaire

A demographic questionnaire collected information about demographic variables, which also included an item for rating self-perceived degree of OSA.

#### The online shopping addiction scale

The preliminary version of the scale included 27 items, each rated on a five-point Likert scale (1 = completely disagree, 2 = disagree, 3 = neither disagree nor agree, 4 = agree, and 5 = completely agree). As can be seen in the result section, the final version consisted of 18 items. Internal consistency (Cronbach's alpha) was 0.90 and 0.95 for the confirmatory and the validation samples, respectively.

#### The compulsive buying scale

The Edward's Compulsive Buying Scale (Ridgway et al., 2008) is a 13-item instrument assessing compulsive buying behavior. Each item was rated on a four-point scale with anchor of 1, with higher scores indicating a tendency toward compulsive buying. Cronbach's alpha was 0.93 for the validation sample.

#### The internet addiction test

This test consists of 20 items, each rated on a five-point Likert scale. High scores on the test indicated serious problems caused by the internet (Young, 1998). Cronbach's alpha was 0.96 for the validation sample.

### Procedure

All three samples were collected online. We distributed the survey link in different students groups at about 30 colleges in China during the winter of 2016 and the spring of 2017. The study was carried out in accordance with the Helsinki Convention and the Norwegian Health Research Act. The protocol and the survey packet were reviewed and approved by the Ethics Committee of the research team's university. The survey could be accessed online for 1 week for each sample. The purpose of the study was displayed on the top of the first webpage of the e-questionnaire. Participants were deemed to be consent to participate if and only if the survey was completed. All questions were collected anonymously and no money or other incentives were given. For both the exploratory and confirmatory samples, the demographic questionnaire and the preliminary OSA scale were posted, while for the validation sample, the Compulsive Buying Scale and the Internet Addiction Test were additionally included.

### Statistics

With sample 1, item discrimination based on classical test theory (CTT) was used to select the most effective items to form a highly reliable and valid instrument. The selected items were then used to explore the possible factor structure with the exploratory factor analysis (EFA). With sample 2, the factor structure explored was justified with the confirmatory factor analysis (CFA). In addition, the psychometric properties and validity evidence of the OSA scale were also assessed via sample 3.

Model fit was evaluated in terms of goodness of fit statistics, specifically the chi-square, comparative fit index (CFI), the Tucker-Lewis index (TLI), root mean square error of approximation (RMSEA), and the standardized root mean square residual (SRMR). Statistical analyses were conducted with Mplus 7.2 (Muthén and Muthén, 2013). The criteria for good fit statistics were non-significant chi-square, CFI ≥ 0.96, TLI ≥ 0. 95, and RMSEA ≤ 0.06 (Hu and Bentler, 1999), and for acceptable fit were CFI ≥ 0.90, TLI ≥ 0 .90, and RMSEA ≤ 0.08 (Vandenberg and Lance, 2000; Marsh et al., 2004). Additionally, SRMR values below 0.08 were typically considered to reflect reasonable model fit (Hu and Bentler, 1999).

## Results

### Scale construction

#### Item analysis

The preliminary scale included 27 items developed with both empirical and theoretical underpinnings. Item assessment was intended to identify items that would be problematic to remain in the following analyses. We considered items with low corrected item-total correlations as problematic and excluded them from subsequent analyses. In addition, we also reviewed the item endorsement frequencies (noting those items whose frequencies fell below 90% or above 10%) to detect whether there was adequate item variance across participants and skewed responses.

As a result, we identified 18 satisfactory items based on Sample 1. Three items were included within each of the six sub-domains to assure content validity. Table 1 showed that the corrected item-total correlation (CITC) of the 18 items ranged from 0.25 to 0.68. Unfortunately, responses were found to depart somewhat from normal distributions, with skewness levels ranging from −0.89 to 2.31.

#### Exploratory factor analysis

Based on Sample 1, the final 18 items were used to conduct EFA. Considering some of the 18 items had non-normal distribution, EFA was run using the robust weighted least square mean and variance (WLSMV) estimation. Item responses were treated as categorical variables, and polychoric correlations were analyzed. CFI, TLI, RMSEA, and SRMR were reported for each factor solution in Table 2. Oblique rotations using the GEOMIN method were generated because the intended OSA factors were correlated.

**Table 2:** **Summary of model fit information for exploratory factor analysis**.

	**Chi-Square**	**df**	**CFI**	**TLI**	**RMSEA**	**90% RMSEA**	**SRMR**	**Chi-Square compared**
1-factor	2390.70^*^	135	0.84	0.82	0.13	[0.125, 0.134]	0.09	
2-factor	952.09^*^	118	0.94	0.93	0.08	[0.079, 0.089]	0.05	846.92^*^(1-factor against 2-factor)
3-factor	686.01^*^	102	0.96	0.94	0.08	[0.070, 0.081]	0.04	245.97^*^(2-factor against 3-factor)
4-factor	441.35^*^	87	0.98	0.96	0.06	[0.058, 0.070]	0.03	212.72^*^(3-factor against 4-factor)
5-factor	281.50^*^	73	0.99	0.97	0.05	[0.047, 0.060]	0.02	139.47^*^(4-factor against 5-factor)
6-factor	194.59^*^	60	0.99	0.98	0.05	[0.040, 0.055]	0.02	83.66^*^(5-factor against 6-factor)
7-factor	121.26^*^	48	1.00	0.99	0.04	[0.030, 0.048]	0.01	68.91^*^(6-factor against 7-factor)

*CFI, comparative fit index; TLI, Tucker-Lewis index; RMSEA, Root Mean Square Error of Approximation; SRMR, Standardized Root Mean Square Residual*;

* *Significant at 5% level*.

Among all the seven solutions EFA extracted, both the six-factor and the seven-factor structure underlies the newly developed OSA scale judging by the criteria of model fit index. Furthermore, the seven-factor is somewhat superior to the six-factor solution from a model comparison perspective. On the one hand, however, including an additional 7th factor in the structure only contributes 3.7% percent of more variance to be explained. On the other hand, from the perspective of substantive theory, the factor loadings pattern for the current six-factor solution shown in Table 3 was nearly the ideal simple theoretical factor structure. Therefore, we finally chose the six-factor structure as the optimal factor structure. In particular, the explored factors could be approximately defined as salience, withdrawal, relapse, conflict, tolerance, and mood modification. As to the six-factor solution, the corresponding eigenvalues for sample correlation matrix were 7.73, 1.81, 1.01, 0.91, 0.83, 0.77, respectively.

**Table 3:** **Exploratory factor analysis factor loadings for the six-factor model of the online shopping addiction scale using weighted least square mean and variance with GEOMIN method rotation**.

**Items**	***f*_1_**	***f*_2_**	***f*_3_**	***f*_4_**	***f*_5_**	***f*_6_**
S1	0.40	0.02	0.15	0.05	−0.01	0.17
S2	0.76	−0.01	0.17	0.09	−0.05	0.00
S3	0.88	0.02	−0.08	−0.05	0.02	0.01
T1	−0.01	0.78	0.03	0.28	0.00	−0.01
T2	0.17	0.27	−0.01	−0.02	0.11	0.45
T3	0.14	0.11	0.07	−0.04	0.46	0.08
M1	0.37	−0.02	0.60	0.01	0.08	−0.19
M2	−0.02	0.04	0.59	−0.21	0.26	0.03
M3	0.05	0.03	0.60	0.03	−0.04	0.22
W1	0.07	−0.02	0.27	0.63	0.03	0.04
W2	0.21	0.16	0.24	0.30	0.15	0.05
W3	−0.05	0.11	0.33	0.50	−0.03	0.04
R1	0.08	−0.09	−0.01	0.42	0.56	−0.08
R2	−0.07	0.04	−0.02	0.39	0.53	0.05
R3	−0.02	0.02	0.07	0.06	0.72	0.15
C1	0.01	0.05	−0.10	0.30	0.05	0.51
C2	−0.07	−0.13	0.07	0.07	0.03	0.53
C3	0.01	−0.01	0.12	0.00	0.02	0.76

Although, the nearly perfect simple six-factor structure was explored, it should be noted that there were still some obvious cross-loading associated with a few items, such as item T2, T3, M1, W3, R1, R2, and C1. For example, item T2 does not only load on tolerance, but also loads high on conflict. Item T3 loads only weak on tolerance, but high on relapse. Similarly, item M1 loads significantly on its theoretical dimension and on salience dimension, and the same pattern occurred to W3, R1, R2, and C1. Additionally, the covariance matrix for the EFA Sample was attached in Appendix.

#### Confirmatory factor analysis

**Figure 1:**
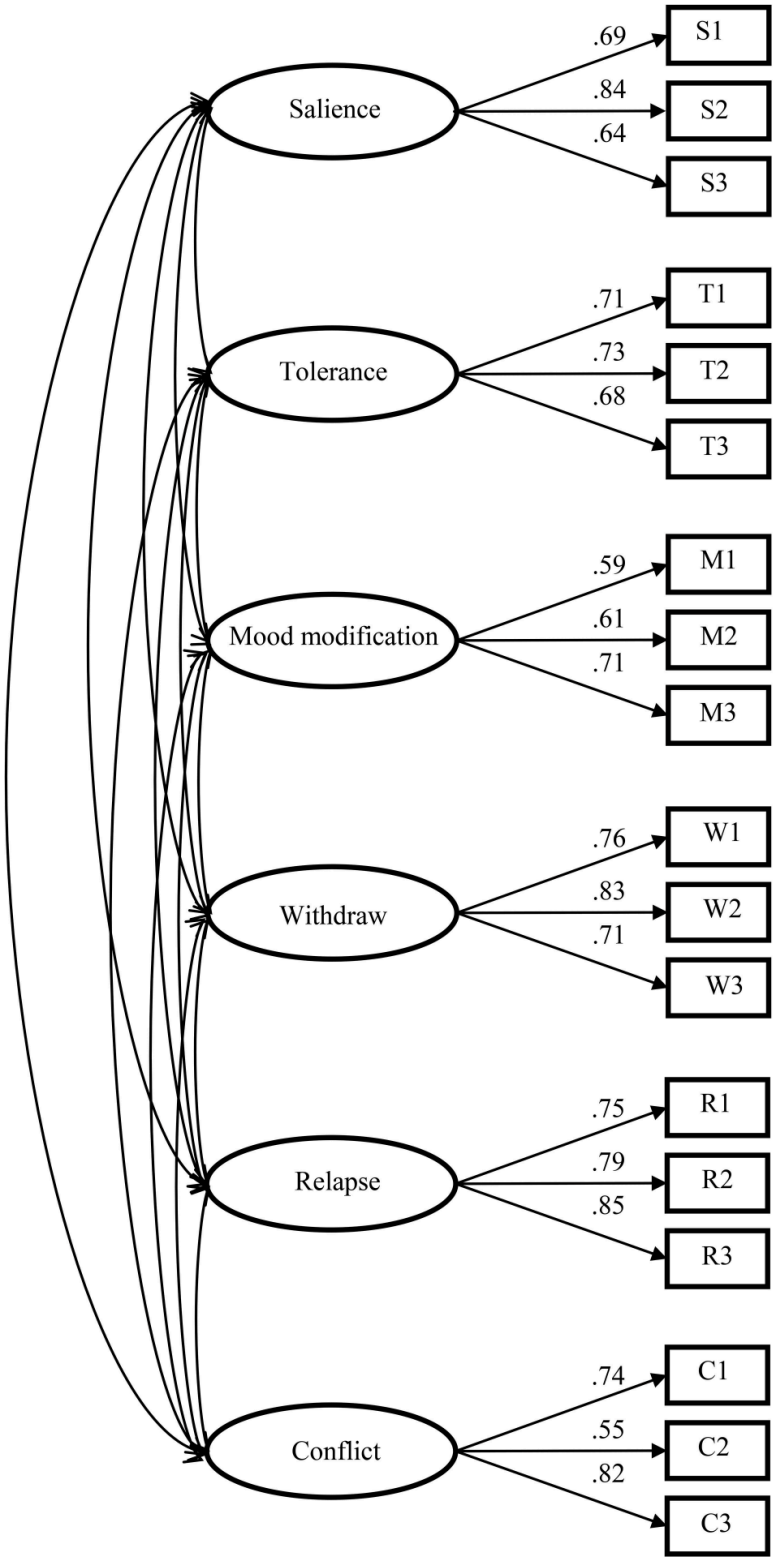
**The six-factor model and its standardized factor loadings**.

Based on the results of EFA and the substantial theory, the six-factor structure was replicated through CFA using weighted least square mean and variance (WLSMV) estimation. For this six factor model in sample 2, the Chi-Square test was 825.22 with degrees of freedom as 120, the CFI was 0.95, the TLI was 0.94, and the RMSEA was 0.08, suggesting acceptable model fit. The corresponding standardized factor loadings of the six-factor model were showed in Figure 1. As can be seen, the factor loadings were high and ranged from 0.55 to 0.84. At the same time, the intercorrelations between six factors was also presented in Table 4, which showed that the six factors were highly correlated. Additionally, the covariance matrix for the CFA Sample was attached in Appendix.

**Table 4:** **The intercorrelations between six factors based on the confirmatory factor analysis**.

**Factors**	***f*_1_**	***f*_2_**	***f*_3_**	***f*_4_**	***f*_5_**	***f*_6_**
*f*_1_	1					
*f*_2_	0.67	1				
*f*_3_	0.77	0.85	1			
*f*_4_	0.68	0.88	0.84	1		
*f*_5_	0.58	0.92	0.76	0.85	1	
*f*_6_	0.39	0.83	0.68	0.73	0.82	1

### Psychometric properties of the online shopping addiction scale

#### Internal consistency

The Cronbach's alpha was 0.95 for samples 3, which indicated a high degree of internal consistency. Table 5 showed that alpha varied between 0.71 and 0.84 for different subscales, indicating that internal consistencies of the most subscales were satisfactory. It should be noted that Cronbach alpha was 0.71 for “Mood modification” subscale and was 0.76 for “Salience” subscale. In view that each subscale consists only three items, these coefficients were acceptable. Inter-correlations between subscales ranged from 0.48 to 0.78, all statistically significant at the 0.01 level.

**Table 5:** **Internal consistencies (Cronbach's alpha) and the inter-correlations for subscales based on the validity Sample**.

**Subscales**	**Alpha**	**1**	**2**	**3**	**4**	**5**	**6**
1 Salience	0.76	1					
2 Tolerance	0.84	0.71	1				
3 Mood modification	0.71	0.67	0.70	1			
4 Withdrawal	0.83	0.68	0.77	0.70	1		
5 Relapse	0.84	0.64	0.77	0.65	0.78	1	
6 Conflict	0.83	0.48	0.67	0.59	0.69	0.71	1

*All correlations were computed with composite scores and were significant at 0.01 level*.

#### Concurrent validity

We assessed Concurrent validity against scores on the Edward's Compulsive Buying Scale (Ridgway et al., 2008) and the Internet Addiction Test (Young, 1998), as external measures of constructs similar to OSA. The evaluation of concurrent validity relies on an understanding of how strongly constructs should or should not relate to each other. Table 6 showed the correlations between the total score of 18-item OSA scale, its subscale scores and the other two scales. Correlations with the CB scale were higher than those with the IA test, which indicated the construct of OSA actually focused not only on excessive shopping behaviors generally, but also fulfill this inclination on the internet.

**Table 6:** **Correlations between the total score and sub-scale scores of the online shopping addiction scale and the scores of the compulsive buying scale and the internet addiction test**.

	**Compulsive buying**	**Internet addiction**
Salience	0.54	0.51
Tolerance	0.69	0.58
Mood modification	0.66	0.55
Withdrawal	0.66	0.57
Relapse	0.67	0.57
Conflict	0.64	0.53
OSA	0.75	0.64

*All correlations were significant at 0.01 level*.

#### Predictive validity

The self-perceived online shopping addiction was an important indicator to assess group differences and practical prediction. Here the item asked the participants to describe their self-perceived degree of OSA in 1 = severe, 2 = moderate, 3 = mild, and 4 = no addiction.

We first assessed the utility of the OSA scale by examining group differences between four responses with variance analysis technique. The homogeneity of group variance was first examined with Levene's test. As was showed in Table 7, the Levene's test was significant and suggested that the group variances were not equal. However, the variance ratio computed with largest variance divided by the smallest one was 2.83, which was too small to worry much about. The group difference was significant at 0.01 level with *F*_(3, 324)_ = 60.92. Finally, all possible pair-wise comparisons of means was conducted using the Least Significant Difference (LSD) test. The *Post-hoc* comparisons showed that the means were ordered as expected.

**Table 7:** **Descriptive statistics for the self-perceived OSA and the test of homogeneity of variance**.

**Self-perceived**	***M***	***N***	***SD***	**Levene's statistic**	**df1**	**df2**	**Sig**.
1	66.91	11	18.99	3.73	3	324	0.01
2	51.74	78	13.28				
3	42.40	134	12.55				
4	30.11	105	11.29				

We further tested the agreement between self-perceived and model-predicted membership via the classification table from logistic regression. We recoded severe and moderate self-ratings as addiction, and the mild and no addiction ratings as non-addiction. The subsequent logistic regression of self-perceived addiction on the item performances yielded the percentage of correctly prediction shown in Table 8. The overall correctly predicted percentage was 79.60, implying the precision of the scale for screening and diagnosis.

**Table 8:** **Consistency of Self-Diagnostic and Predicted Addiction by the OSA Scale**.

		**Predicted addiction**		**Percentage correct**
		**0**	**1**	
Self-perceived addiction	0	220	19	92.10
	1	48	41	46.10
Overall percentage				79.60

## Discussion

We conducted the present study to develop a reliable and valid instrument for OSA. Based on previous research on behavioral addiction, we adopted the widely accepted six-factor component model (Brown, 1993; Griffiths, 1996) and constructed an 18-item OSA scale, with each component measured by three items. The results of EFA indicated that the six-factor structure underlay the newly developed scale from perspectives of model comparison and substantive theory. Moreover, the results of CFA also demonstrated that the six-factor structure fit the data well. In terms of reliability and validity, the Cronbach's alpha suggested that the scale was highly reliable and the concurrent validity was also satisfactory as indicated by correlations between the scale and measures of similar constructs. Finally, the OSA scale scores predicted the self-perceived online shopping addiction to a relative high degree. We conclude that the present 18-item scale is a solid theory-based instrument to empirically measure online shopping addiction.

It has been argued that since the late 1990s that most people who spend excessive time on the internet are not addicted to the medium itself, but use it to fulfill specific addiction, such as video game playing or shopping (Griffiths, 1999, 2000). In order to clarify the structure of OSA, it was necessary to make clear the relationship between generalized and specific IA. The present study held that although specific type had their special objects of thinking, feeling, and activities, they shared common components with generalized type. Consequently, the target of the present study, online shopping addiction, indeed could be represented by the six-factor component model.

The results showed the commonalities between specific internet addiction and generalized internet addiction in that both constructs had the common six components of behavioral addition. However, what is the particularity of online shopping addiction as a type of specific internet addiction? When we examined the content of the items of two validity scales, it was obvious that items of the OSA scale shared more similarities with those of the Internet Addiction Test. However, the analysis of concurrent validity showed that online shopping addition was more relevant to compulsive buying than to internet addiction. This contrast suggested that the similarities between OSA and IA were more superficial than those between OSA and CB. Furthermore, OSA is more than a form of internet addiction. In nature, it is a form of shopping addiction and addicts use the internet mainly to fulfill their problematic shopping inclination. This hinted us that in terms of the diagnosis and intervention of specific internet addiction, more emphasis should be put on its peculiarities.

When we examined the agreement between self-perceived and model-predicted membership, the misclassified ratio was a little high for the self-reported addiction category. This can be partly due to the nature of OSA and the characteristic of the present sample. As a behavioral addiction, the base rate of OSA in non-clinical sample could be rather low, just like the similar disorder, compulsive buying (Black, 2007). Furthermore, it was inevitable for some participants answered the survey in a casual way, especially for an online version. This was possible since a large percent rated themselves as having severe or moderate addiction, which indicated that some participants had the inclination to exaggerate their own status. Therefore, self-perceived degree of OSA could only be a very gross indicator and it finally contributed to the relative high ratio for misclassification.

### Limitations and suggestions for future research

Based on the six-factor component model for behavioral addiction (Brown, 1993; Griffiths, 1996), we constructed a specialized instrument for online shopping addiction and acquired reasonable results. However, the appropriateness of the structure was unable to cover up a couple of deficiencies in the present study. Firstly, although the present scale asked the examinees to respond on a five-point scale, there were still substantial amounts of participants choosing to respond on only four categories or less. It is still unclear why participants choose to response in less categories than demanded. This may result from that it is difficult for participants to distinguish the subtleness of adjacent category when the number of categories amounts to some extent. It may also result from that some participants are just inclined to response in very limited categories, which is a common style under the circumstances of Chinese culture. In the next phase, we plan to change the scale into a few formats to explore the optimal number of response category.

In the present study, the results of EFA did conform to a simple six-factor structure. The factor pattern was clear with only a few items embodying substantial cross-loadings. These cross-loadings could probably due to the nature of the construct. Since different factors were set to be oblique during the EFA process, items pertaining to highly correlating factors could be confused instinctively. Furthermore, when we examined the correlation matrix for exploratory sample thoroughly, it was clear that some item pairs turned out to correlate rather high, such as M1 and S2, C3, and T2, and T3 and R3. These high correlations between items from different factors might be due to phrasing and content of items, which could possible contribute to significant cross-loadings. Cross-loadings can contaminate the structure of the construct, which was especially true for the “tolerance” element. Among the three items of this subscale, there was one item loading rather high on “relapse” and another item loading significantly both on “withdrawal” and “tolerance.” As for these cross-loadings and possible flaw in items, we also plan to do more research to recognize which loadings naturally do not adhere to the structure, and to clarify the structure of construct and the scale.

Although, some studies (Black, 2007; Clark and Calleja, 2008; Lejoyeux and Weinstein, 2010) have shown that shopping addiction and CB overlap to a great extent, we hypothesized that they were distinct, albeit related, constructs. The same principle applied to the relationship between OSA and other behavioral addictions, especially IA. The fact that OSA correlated significantly with CB or IA does not mean that there was causal relationship existing between them, all of which could be the results of more substantial and fundamental factors, such as personality traits (Sun and Wu, 2011; Rose and Dhandayudham, 2014). During the development of the present scale, the similarities between OSA and other behavioral addictions were taken into full consideration, while the peculiarities of OSA still remain to be emphasized in future research.

Additionally, all examinees of the present study were college students, which were rather similar in characteristics and backgrounds. In near future, we plan to distribute the survey in more heterogeneous examinees to acquire more generalized results. Furthermore, new IRT-based methods and techniques would also be implemented in future studies to improve the whole quality of the scale, including the exploration of the elaborated characteristics of the items, the item functioning differences across genders and the measurement invariance across groups.

## Author contributions

TX led the design and implement of the study, including the literature search, analysis, interpretation of the data, drafting, writing, and revising. All authors contributed to the design (HZ, WT, and TX), questionnaires construction and elaboration (HZ, WT, and TX), data collection (HZ and WT), analysis (HZ and WT), interpretation of data (HZ, WT, and TX), and writing and revising the work critically (HZ, WT, and TX). All authors read and approved the final version of the work to be published (HZ, WT, and TX) and agreed to be accountable for all aspects of the work in ensuring that any question to the accuracy of the work is appropriately investigated and resolved (HZ, WT, and TX).

## Funding

The manuscript is supported by National Natural Science Foundation of China (Award number 31371047).

### Conflict of interest statement

The authors declare that the research was conducted in the absence of any commercial or financial relationships that could be construed as a potential conflict of interest.
