# Supplementary material for: The Development and Validation of the Online Shopping Addiction Scale
Source: Front Psychol. 2017 May 16;8:735. doi: 10.3389/fpsyg.2017.00735 (PMC5432625; doi:10.3389/fpsyg.2017.00735)
Supplement: Supplementary file 1 [file DataSheet1.docx]

Appendix

Table 9

*The Covariance Matrix for the Exploratory Factor Analysis Sample.*

|  | S1 | S2 | S3 | T1 | T2 | T3 | M1 | M2 | M3 | W1 | W2 | W3 | R1 | R2 | R3 | C1 | C2 | C3 |
| --- | --- | --- | --- | --- | --- | --- | --- | --- | --- | --- | --- | --- | --- | --- | --- | --- | --- | --- |
| S1 | 1.20 |  |  |  |  |  |  |  |  |  |  |  |  |  |  |  |  |  |
| S2 | .48 | 1.37 |  |  |  |  |  |  |  |  |  |  |  |  |  |  |  |  |
| S3 | .37 | .74 | 1.06 |  |  |  |  |  |  |  |  |  |  |  |  |  |  |  |
| T1 | .30 | .33 | .14 | 1.39 |  |  |  |  |  |  |  |  |  |  |  |  |  |  |
| T2 | .35 | .35 | .24 | .61 | 1.27 |  |  |  |  |  |  |  |  |  |  |  |  |  |
| T3 | .43 | .42 | .31 | .50 | .51 | 1.66 |  |  |  |  |  |  |  |  |  |  |  |  |
| M1 | .44 | .70 | .45 | .36 | .31 | .43 | 1.23 |  |  |  |  |  |  |  |  |  |  |  |
| M2 | .25 | .38 | .18 | .43 | .38 | .49 | .53 | 1.56 |  |  |  |  |  |  |  |  |  |  |
| M3 | .40 | .48 | .24 | .50 | .50 | .44 | .60 | .63 | 1.48 |  |  |  |  |  |  |  |  |  |
| W1 | .39 | .52 | .28 | .53 | .42 | .47 | .47 | .34 | .52 | 1.30 |  |  |  |  |  |  |  |  |
| W2 | .41 | .65 | .38 | .66 | .54 | .66 | .57 | .54 | .61 | .72 | 1.49 |  |  |  |  |  |  |  |
| W3 | .34 | .39 | .22 | .59 | .39 | .47 | .41 | .37 | .58 | .75 | .66 | 1.49 |  |  |  |  |  |  |
| R1 | .35 | .44 | .27 | .42 | .43 | .47 | .39 | .33 | .42 | .63 | .59 | .45 | 1.18 |  |  |  |  |  |
| R2 | .24 | .30 | .18 | .48 | .46 | .45 | .31 | .33 | .41 | .50 | .57 | .50 | .63 | 1.07 |  |  |  |  |
| R3 | .30 | .34 | .23 | .49 | .54 | .68 | .35 | .48 | .48 | .53 | .59 | .49 | .61 | .61 | 1.08 |  |  |  |
| C1 | .21 | .18 | .07 | .33 | .35 | .28 | .11 | .15 | .30 | .35 | .36 | .31 | .32 | .37 | .36 | .78 |  |  |
| C2 | .10 | .07 | -0.01 | .13 | .17 | .17 | .06 | .11 | .17 | .17 | .16 | .19 | .15 | .16 | .23 | .18 | .69 |  |
| C3 | 0.20 | 0.16 | .07 | .31 | .40 | .32 | .16 | .29 | .33 | .33 | .35 | .30 | .27 | .31 | .39 | .29 | .20 | .63 |

Table 10

*The Covariance Matrix for the Confirmatory Factor Analysis Sample.*

|  | S1 | S2 | S3 | T1 | T2 | T3 | M1 | M2 | M3 | W1 | W2 | W3 | R1 | R2 | R3 | C1 | C2 | C3 |
| --- | --- | --- | --- | --- | --- | --- | --- | --- | --- | --- | --- | --- | --- | --- | --- | --- | --- | --- |
| S1 | 1.76 |  |  |  |  |  |  |  |  |  |  |  |  |  |  |  |  |  |
| S2 | .92 | 1.65 |  |  |  |  |  |  |  |  |  |  |  |  |  |  |  |  |
| S3 | .86 | .70 | 1.48 |  |  |  |  |  |  |  |  |  |  |  |  |  |  |  |
| T1 | .83 | .97 | .59 | 1.58 |  |  |  |  |  |  |  |  |  |  |  |  |  |  |
| T2 | .81 | .95 | .51 | .98 | 1.46 |  |  |  |  |  |  |  |  |  |  |  |  |  |
| T3 | .96 | 1.08 | .67 | 1.02 | 1.06 | 1.72 |  |  |  |  |  |  |  |  |  |  |  |  |
| M1 | .72 | .64 | .83 | .61 | .48 | .71 | 1.41 |  |  |  |  |  |  |  |  |  |  |  |
| M2 | .66 | 1.03 | .46 | .70 | .73 | .94 | .67 | 1.68 |  |  |  |  |  |  |  |  |  |  |
| M3 | .71 | .86 | .36 | .89 | .86 | .90 | .59 | .83 | 1.54 |  |  |  |  |  |  |  |  |  |
| W1 | .89 | .82 | .65 | .83 | .74 | .84 | .60 | .62 | .74 | 1.43 |  |  |  |  |  |  |  |  |
| W2 | .74 | 1.09 | .57 | .96 | .98 | 1.06 | .52 | .91 | 1.00 | .86 | 1.52 |  |  |  |  |  |  |  |
| W3 | .74 | .82 | .49 | .95 | .81 | .90 | .61 | .74 | .88 | 1.07 | .89 | 1.58 |  |  |  |  |  |  |
| R1 | .80 | .81 | .62 | .84 | .85 | .90 | .56 | .71 | .73 | 1.03 | .85 | .81 | 1.48 |  |  |  |  |  |
| R2 | .64 | .77 | .50 | .88 | .76 | .79 | .47 | .72 | .72 | .78 | .81 | .91 | .89 | 1.39 |  |  |  |  |
| R3 | .70 | .90 | .45 | .89 | 1.00 | .96 | .47 | .79 | .81 | .74 | .92 | .80 | .91 | .87 | 1.33 |  |  |  |
| C1 | .47 | .55 | .31 | .62 | .65 | .62 | .31 | .53 | .64 | .60 | .60 | .68 | .64 | .70 | .62 | .96 |  |  |
| C2 | .33 | .43 | .17 | .51 | .56 | .48 | .20 | .46 | .56 | .45 | .61 | .57 | .48 | .45 | .57 | .56 | .86 |  |
| C3 | .55 | .72 | .35 | .70 | .82 | .74 | .35 | .72 | .70 | .68 | .75 | .75 | .70 | .62 | .87 | .67 | .62 | 1.13 |
